# Left-sided acute appendicitis in a patient with situs viscerum inversus totalis: A case report

**DOI:** 10.1016/j.ijscr.2021.106658

**Published:** 2021-12-04

**Authors:** Giuseppe Evola, Francesco Ferrara, Giovanni Francesco Di Fede, Marco Patanè, Salvatore Sarvà, Luigi Piazza

**Affiliations:** a General and Emergency Surgery Department, Garibaldi Hospital, Piazza Santa Maria di Gesù 5, 95100 Catania, Italy; b Department of Radiology, Santa Marta e Santa Venera Hospital, Via Caronia, 95024 Acireale, Catania, Italy

**Keywords:** Left-sided acute appendicitis, Situs viscerum inversus totalis, Left lower quadrant pain, Emergency surgery, Case report

## Abstract

**Introduction and importance:**

Left-sided acute appendicitis (LSAA) is a very rare cause of acute abdomen, developing in association with two types of congenital anomalies like as situs viscerum inversus (SVI) and midgut malrotation (MM). Preoperative diagnosis of LSAA is a challenge because of its rarity and atypical presentation. Imaging may be helpful for determining the correct diagnosis. Surgery represents the standard treatment of LSAA.

**Case presentation:**

A 67-year-old Caucasian male with presented to the Emergency Department with a two-day history of left lower quadrant (LLQ) abdominal pain, nausea, vomiting, diarrhea and fever. Physical examination revealed LLQ abdominal rebound tenderness with guarding. Laboratory tests reported high levels of C-reactive protein and neutrophilic leukocytosis. Abdominal contrast-enhanced computed tomography showed a LSAA with intraluminal appendicoliths, fat infiltration and pericecal fluid collection in a patient with SVI. The patient underwent laparoscopic appendectomy: a gangrenous and perforated appendicitis was sectioned and removed with drainage of pericecal abscess. The postoperative course of the patient was uneventful.

**Clinical discussion:**

LSAA is characterized by anatomical variation of appendix and atypical presentation. Preoperative clinical diagnosis of LSAA is very difficult and imaging may be helpful for determining the correct diagnosis, as well as confirming SVIT or MM. Laparoscopic appendectomy represents the correct treatment of LSAA.

**Conclusion:**

LSAA is a rare surgical emergency that should be considered in the differential diagnosis of patients with LLQ abdominal pain. Preoperative diagnosis of LSAA needs a high index of suspicion and is facilitated by imaging. Surgery represents the appropriate treatment of LSAA.

## Highlights

• Left-sided acute appendicitis (LSAA) is a very rare cause of acute abdomen.
• LSAA develops in association with situs viscerum inversus or midgut malrotation.
• Diagnosis of LSAA is a challenge because of the absence of specific clinical presentation.
• Laparoscopic appendectomy is the standard treatment of LSAA.

## 1 Introduction

Acute appendicitis (AA) represents one of the most common abdominal diseases requiring emergency surgery and accounting for 4–8% of all emergency department visits [1]. Diagnosis of AA is usually relative simple and is based on clinical symptoms, physical examination and radiology, however the malposition or anatomical variation of the appendix make it uncertain and can delay the surgical treatment favoring the onset of complications such as abscess formation, perforation or peritonitis. Left-sided acute appendicitis (LSAA), characterized by anatomical variation of appendix and atypical presentation, develops in association with two types of congenital anomalies like as situs viscerum inversus (SVI) and midgut malrotation (MM). LSAA is still an easily missed diagnosis and its management is the same as for normally sited acute appendicitis, however delayed diagnosis of LSAA often requires surgery. A rare case of LSAA in a patient with situs viscerum inversus totalis (SVIT), managed in emergency by laparoscopic surgery, is presented with review of the literature in accordance with SCARE 2020 criteria [2]. The purpose of this case report is to remember that LSAA is a very rare cause of acute abdomen that may require emergency surgery.

## 2 Presentation of case

**Fig. 1:**
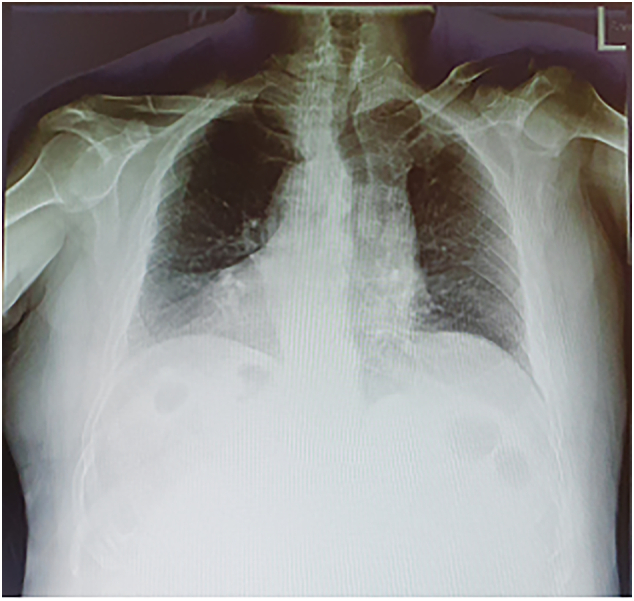
Chest X-ray showing dextrocardia.

**Fig. 2:**
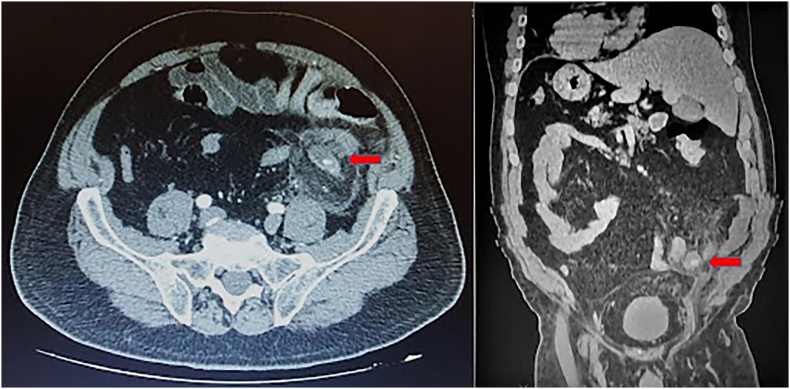
A, B. Preoperative abdominal CECT confirming SVIT and showing LSAA with intraluminal appendicolithis (red arrow). A transverse view, B coronal view. (For interpretation of the references to colour in this figure legend, the reader is referred to the web version of this article.)

**Fig. 3:**
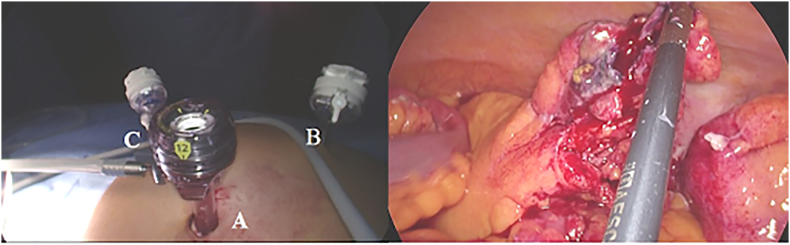
A,B. Panel A: Placement of three 12-mm trocars: in the umbilical region (A), in the right flank (B) and in the right iliac fossa (C). Panel B: Left-sided acute gangrenous and perforated appendicitis: operative findings.

**Fig. 4:**
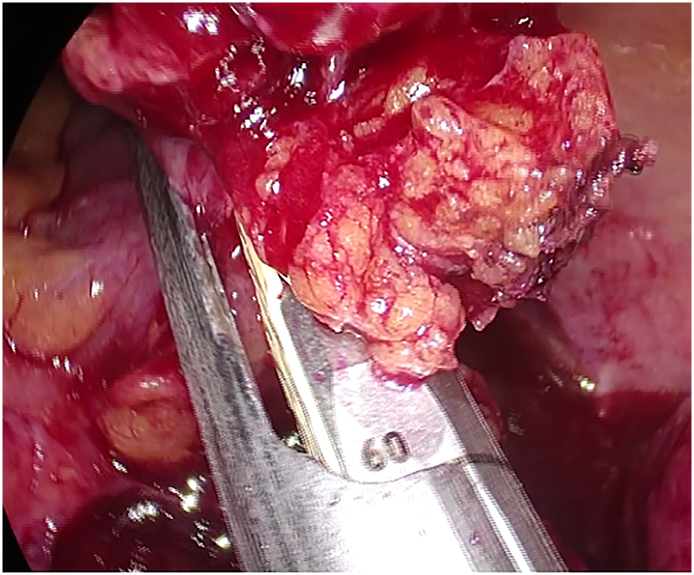
Section of the appendix with mechanical stapling device.

**Fig. 5:**
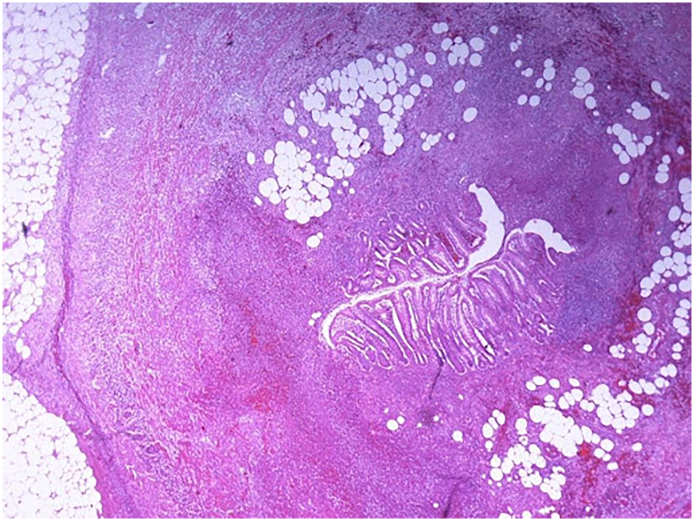
Photomicrograph section of acute appendicitis (haematoxylin and eosin, original magnification x 20).

A 67-year-old Caucasian male presented to the Emergency Department with a two-day history of left lower quadrant (LLQ) abdominal pain, nausea, vomiting, diarrhea and fever (38 °C); others vital signs were normal. The patient was aware of having SVIT and was on hypertensive medications for ten years. He referred habit on smoking but denied alcohol consumption, his familial medical history was normal. The patient was retired from the work, married and of medium socio-economic status. Physical examination revealed mild abdominal distention, LLQ abdominal rebound tenderness with guarding and hypoactive bowel sound. Laboratory tests reported high levels of C-reactive protein (135.5 mg/L) and neutrophilic leukocytosis (WBC 15.700 10^3^/μL). The patient was initially managed with fluids, intravenous broad-spectrum antibiotics and bowel rest. A chest X-ray confirmed dextrocardia (Fig. 1). After a plain abdominal radiography, the patient was subsequently evaluated by abdominal contrast-enhanced computed tomography (CECT) which confirmed SVI and revealed an inflamed and dilated left-sided appendix with intraluminal appendicoliths, fat infiltration and pericecal fluid collection (Fig. 2A, B). The patient, after understanding the severity of his medical condition and accepting surgery, was taken emergently to the operating room by experienced general surgeons for laparoscopic appendectomy under general anesthesia. On the operating room we placed the monitor on the left side while the surgeon and the cameraman assistant were on the right side of the patient. After induction of pneumoperitoneum with the Veress needle and placement of three 12-mm trocars (in the umbilical region, right flank and right iliac fossa) we explored the peritoneal cavity with evidence of left-sided acute gangrenous and perforated appendicitis with pericecal abscess (Fig. 3A, B) in SVI. After dissection of mesoappendix and cauterization of appendicular artery with bipolar forceps, the appendix was sectioned after using mechanical stapling device (Fig. 4) and removed in an endobag. After drainage of pericecal abscess, washing and aspiration of peritoneal cavity a pericecal drain was placed. Patient was given an IV injection of Amoxicillin/Clavulanate 2 g twice daily and Metronidazole 500 mg thrice daily for five days and a SC injection of enoxaparin sodium 4.000 IU once daily for 21 days. The postoperative course was uneventful: abdominal drain was removed on the 4th postoperative day and laboratory tests were unremarkable. The patient was discharged on the 5th postoperative day in a stable condition. Histopathological examination confirmed acute gangrenous appendicitis (Fig. 5). The patient tolered the advice provided and after a follow-up of six months is asymptomatic.

## 3 Discussion

This clinical case describes a rare LSAA causing acute abdomen in a patient with SVIT. True LSAA occurs in association with two types of congenital anomalies like as SVI and MM. SVI is a rare autosomal congenital defect characterized by the transposition of abdominal and/or thoracic organs: this condition may be totalis (SVIT) when the organs in both thoracic and abdominal cavities are transposed or partial when only one of these cavities is affected. SVIT commonly occurs with dextrocardia and rarely with levocardia [3] and may be associated with multiple congenital defects and vascular anomalies [4]; its incidence varies from 0.001% to 0.01% [5]. MM is caused by nonrotation or incomplete rotation of the primitive intestinal loop around the axis of superior mesenteric artery during fetal life and can manifest itself generally in the first month of life with bowel dysfunction and bilious vomiting, but in most of the cases it, like as SVIT, remained asymptomatic. The incidence of MM anomalies varies from 0.03% to 0.5% in the live births [6]. LSAA may also present as an atypical presentation of a long right-sided appendix projecting into the left lower quadrant [7]. Collins in a study of 71.000 patients with appendicitis, reported that the overall incidence of true LSAA was 0.04%, which included 0.024% with MM and 0.016% with SVIT [8]. According to literature data, LSAA occurs between 8 and 82 years and is 1.5 fold more frequent in men than in women [9]. Diagnosis of AA is based on well-established clinical symptoms and signs, radiologic findings and surgeon experience. In its typical presentation (60% of cases), AA begins with a vague abdominal discomfort around the epigastric or periumbilical region accompanied by nausea and vomiting; several hours later the pain migrates to the right lower quadrant of the abdomen near McBurney's point [10]. Additionally, fever, rebound tenderness, Rovsing's sign, psoas sign, diarrhea and anorexia may be observed. However one third of patients with AA complains abdominal pain in an unexpected location [11], due to the various anatomical position of the appendix [12]. Concerning the abdominal pain localization of LSAA, Akbulut et al. reported that 62.1% of the patients presented with LLQ pain, 14.7% with right lower quadrant pain, 7.3% with bilateral pain, 7.3% with left-upper quadrant pain, 6.3% with peri-umbilical pain and 2% with pelvic pain [9]. It can be explained because of the nervous system may not show the corresponding transposition of the viscera resulting in confusing pain localization with preoperative diagnosis of LSAA only in 51% of the patients [9]. Symptoms of AA may be also caused by rare appendiceal neoplasms [13]: Akbulut reported that only two of 95 patients, who underwent appendectomy due to LSAA, were pathologically diagnosed with malignancy [9]. Because of the unusual displacement of the abdominal viscera in SVI, the diagnosis of LSAA presenting with LLQ abdominal pain can be challenging. The main differential diagnosis of LLQ abdominal pain includes sigmoid diverticulitis, intestinal perforation or obstruction [14], incarcerated or strangulated hernia, regional enteritis, Meckel's diverticulitis, acute pancreatitis, atypical right-sided appendicitis, LSAA, epiploic appendagitis, abdominal aortic aneurysm, mesenteric ischemia, renal colic, psoas abscess, testicular or ovarian torsion, ruptured ovarian cyst, ectopic pregnancy, pelvic inflammatory disease [1]. Imaging may be helpful for determining the correct diagnosis, as well as confirming SVIT or MM. The detection of dextrocardia on chest X-ray, left-sided liver and right-sided gastric bubble on abdominal plane X-ray are of considerable value in establishing the diagnosis of SVIT. Abdominal ultrasound may be useful in locating the position of the inflamed appendix (accuracy of 71%–97%). CECT is superior to others imaging modalities in detection of AA (accuracy of 90%–98%) and should be used to prevent any type of misdiagnosis. In our patient with known SVIT we suspected a LSAA and conducted CECT to confirm the diagnosis. Laparoscopy may be very useful both in establishing the differential diagnosis and in performing the definitive surgery [15]. LSAA represents an increasing cause of misdiagnosis of AA (reported to be high as 24%) [16], leading to abscess formation, perforation of appendix and peritonitis. After establishing the diagnosis of LSAA the surgical options are the same as for normal patients. Laparoscopic appendectomy represents the standard treatment of AA, although intravenous antibiotics may be considered first-line therapy in selected patients. After radiological diagnosis of AA with intraluminal appendicoliths we decided for laparoscopic appendectomy. In literature only few cases of laparoscopic approach in LSAA are reported and the first laparoscopic appendectomy was performed in 1998 by Contini et al. [17]. The laparoscopic technique used in our case report was conventionally with tree three ports, however single-incision multiport appendectomy for a patient with SVIT has been reported [18]. AA is still one of the most common surgical emergencies with low morbidity and mortality if surgical treatment is not delayed. Mortality increases if surgical treatment is delayed [19], often caused by misdiagnosis of AA. The mortality rate of AA is reported to be less than 1%, but it can be increased up to 5% in delayed diagnosed AA [4].

## 4 Conclusion

LSAA represents a rare surgical emergency that should be considered in the differential diagnosis of patients with LLQ abdominal pain. Its diagnosis is a challenge because of the absence of specific clinical presentation and needs a high index of suspicion. Imaging and/or laparoscopy are helpful in establishing the differential diagnosis of LLQ abdominal pain and in detecting LSAA as well as confirming SVIT or MM. Laparoscopic appendectomy is the standard treatment of LSAA.

## Sources of funding

This research did not receive any specific grant from funding agencies in the public, commercial, or not-for-profit sectors.

## Ethical approval

Ethical approval has been exempted by our institution because this is a case report and no new studies or new techniques were carried out.

## Consent

Written informed consent was obtained from the patient, for publication of this case report and accompanying images. A copy of the written consent is available for review by the Editor-in-Chief of this journal on request.

## Registration of research studies

Not applicable.

## Guarantor

Giuseppe Evola.

## Provenance and peer review

Not commissioned, externally peer-reviewed.

## CRediT authorship contribution statement

Giuseppe Evola: Operated on the patient, drafting the manuscript, literature research.

Francesco Ferrara: Operated on the patient, drafting the manuscript.

Giovanni Francesco Di Fede: Drafting the manuscript, literature research.

Marco Patanè: Operated on the patient and literature research.

Salvatore Sarvà: Drafting the manuscript and literature research.

Luigi Piazza: Revising the manuscript.

## Declaration of competing interest

The authors have no conflict of interest to declare.
